# CpGDB : A Comprehensive Database of Chloroplast Genomes

**DOI:** 10.6026/97320630016171

**Published:** 2020-02-29

**Authors:** Bhupinder Pal Singh, Ajay Kumar, Harpreet Kaur, Harpreet Singh, Avinash Kaur Nagpal

**Affiliations:** 1 I.K. Gujral Punjab Technical University, Kapurthala, 144 603, India; 2 Centre for I.T. Solutions, Guru Nanak Dev University, Amritsar, 143005, India; 3 Electronics and Communication Engineering Department, Beant College of Engineering and Technology, Gurdaspur, 143521, India; 4 Department of Botanical and Environmental Sciences, Guru Nanak Dev University, Amritsar, 143005, India; 5 Department of Bioinformatics, Hans Raj Mahila Maha Vidyalaya, Jalandhar, 144008, India

**Keywords:** Chloroplast Genomes, Gene Sequences, Database

## Abstract

Chloroplast Genome Database (CpGDB) is user friendly, web-based, freely available and dynamic relational database which provides a platform for researchers to search and download complete chloroplast genome sequences, individual gene sequences and feature records of plant species belonging to same or different families of spermatophytes. Presently, the database consists of genome sequences, individual gene sequences and feature records of chloroplast genomes of 3823 plant species belonging to 1527 genera from 256 families, which will be updated regularly with the availability of new sequences at NCBI. Extensive data mining of feature records from GenBank files, uniform nomenclature for majority of genes, enriched intron/exon feature records makes CpGDB a valuable resource for studies in chloroplast genomics while complementing existing chloroplast databases.

## Background

Chloroplasts are photosynthetic intracellular organelles of plants and are considered as Earth's main solar energy converters. Besides photosynthesis, chloroplasts are also involved in synthesis of amino acids, vitamins, lipids, pigments, precursors of plant hormones etc. They contain their own distinct genome, which is known to be derived from a cyanobacterial ancestor. Among three genomes of a plant cell, chloroplast genome is the most gene dense constituting more than 100 genes in genome size of 120 to 210 kb [1]. The chloroplast genome generally has a highly conserved organization in term of size, structure and gene content [2]. The presence of a pair of inverted repeats (IRs) is one of the outstanding features of chloroplast genome, which separates two single copy DNA regions, a large single-copy (LSC) and a small single-copy (SSC) region, on a single circular DNA molecule [3].

Recent advances in sequencing techniques have led to a significant increase in the availability of large number of chloroplast genome sequences. Currently, about 4000 eukaryotic chloroplast genomes have been sequenced completely with the best representation from flowering plants [4]. During the past two decades, few attempts have been made to develop specialized databases for chloroplast genomes such as GOBASE (Organelle Genome Database) [5], ChloroplastDB (Chloroplast Genome Database) [6], Database of PCR Primers of Chloroplast Genomes [7] and PCIR (Database of Plant Chloroplast Inverted Repeats) [8]. However, these databases are either non-functional or very much focused to cover only specific aspect of genomic information. The Organelle Genome Database available at NCBI does give information on chloroplast genomes of different plant species but it is more generalized database where annotations for some gene names and features are not uniform. Moreover, these databases does not allow family wise search of chloroplast genomes thereby necessitating the development of a specialized database of chloroplast genomes with enhanced user friendliness.

Chloroplast Genome Database (CpGDB) is an attempt to organize and integrate enormous amount of data related to chloroplast genomes of spermatophytes. It has two levels of information: NCBI based sequence data and curated annotations. Its main focus is to make available complete chloroplast genome sequences and their different features e.g. gene, CDS, rRNA, tRNA, intron, exon etc. for different types of analysis. This is a dynamic relational database available at http://www.gndu.ac.in/CpGDB and supports different queries specifically with respect to comparative genome analysis across different species/genera/families. It allows search by family, genus and gene names along with provision to download complete chloroplast genome sequences or individual gene sequences for selected plant species belonging to same or different families. Additionally, it provides unified annotations with respect to gene names. This database would be a valuable platform for researchers in the field of chloroplast genomics.

## Materials and Methods:

### Data mining and organization

**Figure 1:**
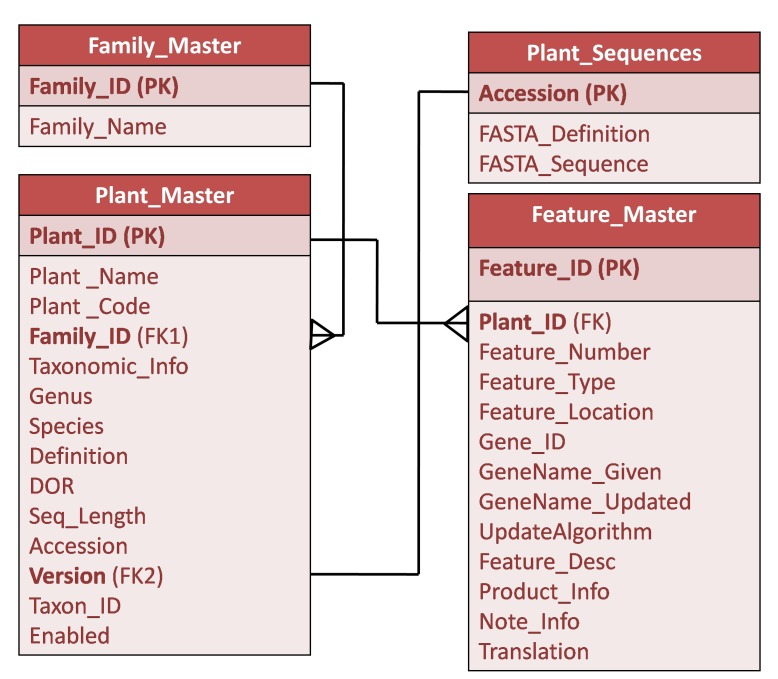
Entity-Relationship diagram of CpGDB.

The CpGDB was designed using MS-Visual Studio 2015 as front end and SQL Server 2012 as back end (Figure 1). A web interface was developed to import complete chloroplast genome sequences belonging to spermatophytes directly into CpGDB from NCBI RefSeq nucleotide database [7] using Entrez Programming Utilities (E-Utilities). The Entrez text query "(Chloroplast [Title] OR Plastid[Title]) AND complete[Title] AND genome[Title] AND refseq[filter] AND plants[filter] AND Spermatophyta[Organism]" was passed as web request to NCBI nucleotide database to download sequences both in FASTA and GenBank format and store in local repository for providing faster and efficient sequence retrieval system.

The GenBank files were parsed using parsing script written in ASP.NET and database tables Family_Master, Plant_Master and Plant_Sequences were updated for each accession. In order to avoid duplicate occurrences of chloroplast genome sequences due to availability of more than one accession for some of the plant species, the most recently modified versions were considered. Also accessions containing hybrid plant species or with missing species name were not considered. Hence, out of total 3881 accessions available upto 30/11/2019, 3823 were considered for further analysis (Supplementary Table S1 - see excel file). These accessions belong to 1527 different genera from 256 plant families. Each plant species was assigned a unique four letter code to make data retrieval, analysis and comparison simple and more convenient. An algorithm was developed to extract and export feature records of plant species from GenBank files. The data related to gene name, geneID, location, product and note information etc. was extracted and stored separately for each plant species. The execution of this algorithm resulted in compilation of 10,56,377 feature records belonging to 3823 plant species. Any missing information was indicated with '-' value in the record.

### Data cleaning:

It is important to mention here that gene names in most of the downloaded GenBank files were not uniform. For example 16S ribosomal RNA was mentioned as rrn16, 16rrn, 16s, 16S ribosomal RNA, 16S rRNA, 16S-rRNA etc. among various chloroplast genomes. To bring uniformity in gene names, an algorithm was developed to determine frequency of different gene names in the database. Out of 781 gene names, 461 gene names were replaced with most frequently used gene names. For example gene names for 16S ribosomal RNA were updated with most frequently used gene name 'rrn16'. In total, 40,274 corrections were made across 979 plant species and a record of changes was also maintained (Supplementary Table S2-see excel file).

**Figure 2:**
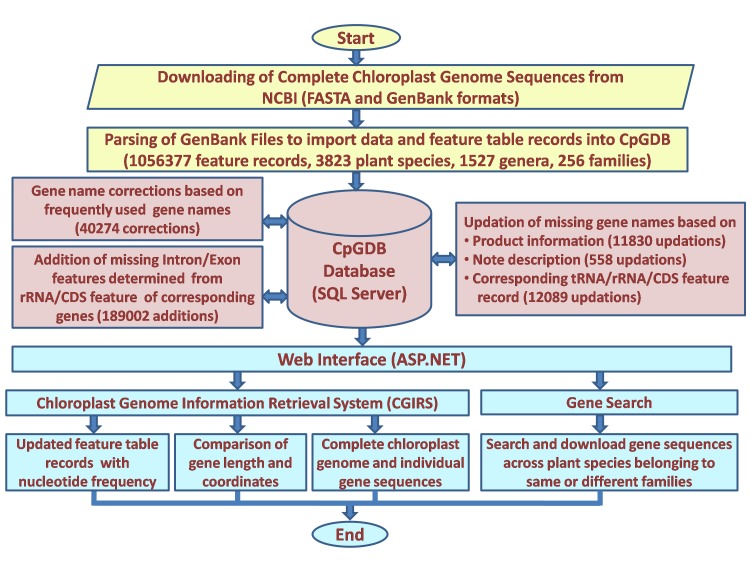
Complete workflow for creation of CpGDB.

A total of 11,830 feature records across 1160 plant species were updated based on their product information given in the feature description due to nonavailability of gene names in the feature records (Supplementary Table S3-see excel file). Similarly, 558 records spanning 115 plant species with missing gene name as well as product information were updated using note description of the corresponding record (Supplementary Table S4-see excel file). Finally, 12,089 records belonging to 1171 plant species with missing gene name, product and note description were updated from the corresponding tRNA/rRNA/CDS feature records (Supplementary Table S5-see excel file). To enrich information about insufficiently annotated split genes, 1,89,002 missing exon/intron features belonging to 3802 plant species were determined using tRNA/CDS features of the corresponding genes and added in the database (Supplementary Table S6-see excel file). All these manual curations resulted in 12,45,379 feature records available in the database. Figure 2 shows the complete workflow along with data for creation of CpGDB.

### Database Interface and Utility:

**Figure 3:**
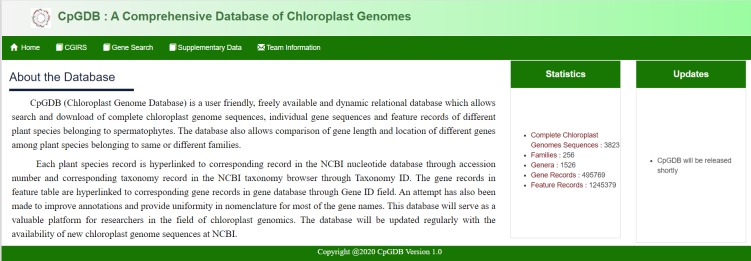
Screen shot showing home page of CpGDB

The database can be accessed easily through Chloroplast Genome Information Retrieval System (CGIRS) link available on the top menu bar of home page of CpGDB (Figure 3).

CGIRS provides a tabular view of families along with list of genera and corresponding species whose chloroplast genome records are available in the database. These records can be filtered and sorted out as per number of genera and number of species. The web interface allows selection of one or more families and/or plant species using check boxes provided against each record. The web interface also provides updated feature records of individual plant species along with the pre-determined nucleotide frequency. The feature records can be filtered based on feature type (All features, Gene, CDS, tRNA, rRNA etc.). The plant species, taxonomy and gene records are hyperlinked to corresponding records in the nucleotide core [9] taxonomy [10] and gene [11] databases, respectively at NCBI. Specific details are displayed on hovering mouse over the plant species name and feature type records. Feature table data as well as chloroplast genome sequences can be downloaded for further analysis. Additionally, user can select multiple plant species and compare lengths and coordinates of all genes across selected plant species. 'Gene Search' link gives an option to select a feature type corresponding to a given gene and download the related sequences or records for selected species.

## Conclusions:

CpGDB provides curated information on complete chloroplast genome sequences available upto 30/11/2019. The database will be regularly updated with the availability of new sequences. The strength of the database lies in its user friendliness, ability to retrieve specific data and uniform nomenclature of majority of genes. CpGDB will serve as a valuable resource for chloroplast genomic studies.

## Supplementary Data:

Supplementary files are available for downloading at CpGDB website.
